# Risk Factors for Post-infectious Bronchiolitis Obliterans in Children: A Systematic Review and Meta-Analysis

**DOI:** 10.3389/fped.2022.881908

**Published:** 2022-06-09

**Authors:** Die Liu, Jing Liu, Lipeng Zhang, Yuanmei Chen, Qi Zhang

**Affiliations:** ^1^Department of Pediatrics, China-Japan Friendship Hospital, Beijing, China; ^2^Graduate School of Peking Union Medical College, Chinese Academy of Medical Sciences, Beijing, China

**Keywords:** meta-analysis, post-infectious bronchiolitis obliterans, bronchiolitis obliterans, risk factors, children

## Abstract

**Background:**

Post-infectious bronchiolitis obliterans (PIBO) is a long-term sequela after an initial insult to the lower respiratory tract. A comprehensive understanding of the factors that contribute to a high risk of developing PIBO is important to help define therapeutic strategies and improve prognosis.

**Methods:**

We performed a systematic review of published literature available in the online databases including PubMed, Embase, Web of Science, CNKI, Wan Fang, and VIP, with the last search updated on 27 January 2022. Observational studies and case-control studies that provide sufficient data to examine associations between potential risk factors and PIBO were included. Pooled odds ratio (OR) or mean difference (MD) with 95% confidence interval (CI) and heterogeneity were calculated.

**Results:**

A total of 14 risk factors were selected from 9 studies included in the analysis. The strongest risk factors were hypoxemia, mechanical ventilation, tachypnea, and wheezing. Hypoxemia conferred the greatest risk with pooled OR of 21.54 (95% CI: 10–46.36, *p* < 0.001). Mechanical ventilation ranked second (pooled OR 14.61, 95% CI: 7.53–28.35, *p* < 0.001). Use of γ-globulin, use of glucocorticoids, co-infection of bacteria, a history of wheezing, and being male were other prominent risk factors. The effects of premature birth, allergic rhinitis, and imaging finding (pulmonary consolidation, atelectasis, pleural effusion) are less clear and require further confirmation. Cases that developing PIBO had a lower age compared with controls (MD, −8.76 months, 95% CI: −16.50 to −1.02, *p* = 0.03). No significant differences were observed in the duration of fever (MD, 1.74 days, 95% CI: −0.07 to 3.54, *p* = 0.06). Children diagnosed with PIBO had higher LDH levels (MD, 264.69 U/L, 95% CI: 67.43 to 461.74, *p* = 0.008) and duration of hospitalization (MD, 4.50 days, 95% CI: 2.63 to 6.37, *p* < 0.001).

**Conclusion:**

In this study, we found that the strongest risk factors for PIBO were hypoxemia, mechanical ventilation, tachypnea, and wheezing. Use of glucocorticoids, γ-globulin, co-infection of bacteria, a history of wheezing, and being male may also play a role. The factors discussed above can inform the generation of a clinical prediction model for the developing PIBO in children.

## Introduction

There are three forms of bronchiolitis obliterans (BO) seen by pediatricians: post-infectious bronchiolitis obliterans (PIBO); BO post lung transplantation; and BO after bone marrow transplantation (BMT) or hematopoietic stem cell transplantation (HSCT) (1, 2). Of these, PIBO is, by far, the most common in children. PIBO is a chronic small-airway disease caused by initial insult to the lower airways and subsequent epithelial and subepithelial inflammation and fibrotic narrowing of the bronchioles (3–5). The injury to the lower respiratory tract can be caused by various pathogens, such as adenovirus, influenza, parainfluenza, respiratory syncytial virus, mycoplasma pneumonia, or measles (6–10).

The prognosis of PIBO is overall poor, which is related to the late diagnosis, irreversible pulmonary fibrosis, and airway obstruction (1, 11). To date, there were several attempts to investigate an association between various risk factors and PIBO in children, but no systematic reviews of published literature assessed the strength of association between the suspected risk factors and PIBO.

Therefore, it is extremely necessary to identify the risk factors predicting the occurrence of PIBO following lower respiratory tract infections. A better understanding and knowledge of the topic can alert the professionals to the need of designing effective strategic measures to reduce the incidence, take early intervention, and improve the prognosis of PIBO in children. This study aimed to assess the quality of available evidence and present summary estimates of the strength of association between the risk factors and PIBO in children using meta-analysis.

## Materials and Methods

This meta-analysis was conducted and reported according to MOOSE guidelines.

### Literature Search

The PUBMED, EMBASE, Web of Science, and Chinese databases (Wan fang, CNKI, and VIP) were systematically searched with the restriction of language only in English and Chinese (last search updated on 27 January 2022). We used the Medical Subject Headings (MeSH): “bronchiolitis Obliterans” as the core search term in case of some unexpected missing of any potentially eligible studies. The references list of each of the initially included studies was also checked for further articles of interest. The final search strategies of PubMed, Web of Science, and Embase are presented in online Supplementary Material.

### Inclusion and Exclusion Criteria

Criteria for inclusion: (1) case-control study, retrospective or prospective observational study, cross-sectional study, and cohort study; (2) all subjects were children (aged 0–18 years) diagnosed with or without PIBO after lower respiratory tract infections; (3) the definition of PIBO is clear; (4) the effect estimates of potential risk factors for PIBO are reported; and (5) sufficient data were provided to calculate odds ratio (OR). Exclusion criteria were as follows: (1) case reports, reviews, or unpublished literature; (2)BO caused by lung transplantation, heart-lung transplantation, HSCT, BMT, and Stevens–Johnson Syndrome; (3)Irrelevant reporting topics: such as organizing pneumonia, interstitial lung disease, bronchiectasis, and cystic fibrosis; and (4) adequate information could be obtained for categorical variables and continuous variables.

### Data Extraction

Data were independently extracted by JL and LZ; any inconsistency was resolved by QZ after referring to the original article. The following variables were collected and recorded in an Excel table: the first author, publication year, region, sample size, age, and risk factors were included. When full data cannot be obtained from the study, we tried to contact the corresponding author to obtain all the data.

### Quality Assessment

The Newcastle–Ottawa Scale (NOS) for case-control studies was used to assess study quality concerning cases and controls selection (four items), comparability (one item), and exposure (three items). The star system ranges from 0 to 9, with higher scores representing better study quality. JL and DL accomplished this process independently, and disagreements were resolved by QZ.

### Statistical Analysis

Statistical analysis was performed using the Review Manager 5.4 software (The Nordic Cochrane Collaboration, Copenhagen). Raw data of continuous variables were converted into mean and standardized difference (SD) when suitable (12). Heterogeneity was tested by the *I*^2^ statistic, and *I*^2^ > 50% was considered significant. If there was significant heterogeneity, a random-effects model was used, or else, a fixed-effects model. The pooled effect estimates were reported as an OR with 95% confidence intervals (CI) for dichotomous data and mean difference (MD) with 95% CI for continuous outcomes. The significance of the pooled effects was determined by the *Z*-test, two-tailed *p* < 0.05 was considered statistically significant.

## Results

### Study Selection

**FIGURE 1:**
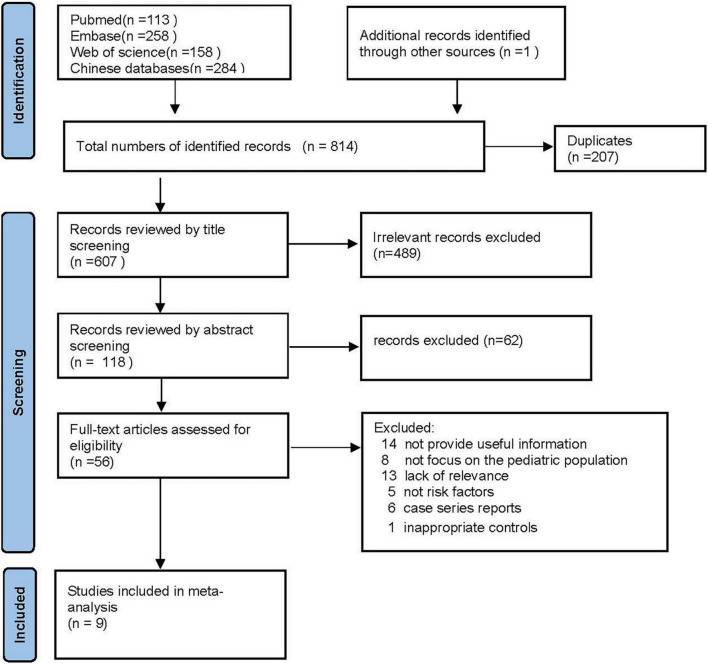
A flow diagram demonstrating the process of inclusion and elimination of studies.

The flowchart of the study selection process is shown in Figure 1. Of 814 articles initially searched, 207 duplicates were firstly removed. After screening the titles and abstracts, 551 studies that were irrelevant to the risk factors of PIBO were excluded, and another 47 studies that did not provide detailed origin data or were not in accordance with our inclusion criteria were excluded after assessing the full text. Finally, only nine studies satisfied our inclusion criteria and were enrolled in our meta-analysis, including three case-control studies, five retrospective studies, and one prospective study.

### Study Characteristics and Study Characteristics

The nine studies included in the qualitative analysis comprised 377 cases and 1,342 controls. The general characteristics were summarized in Table 1. Except for two South America (Chile, Argentina) studies, all the others were on the Asian population (China and Korea). A total of 14 risk factors were eligible for meta-analysis: male, premature birth, a history of wheezing, allergic rhinitis, wheezing, tachypnea, hypoxemia, pulmonary consolidation, atelectasis, pleural effusion, co-infection of bacteria, use of glucocorticoids, use of γ-globulin, and mechanical ventilation. Forest plots for individual risk factors for PIBO are available in Supplementary Figures 1–14. A summary of the pooled OR for all risk factors is given in Table 2. Based on the NOS quality assessment, all the included studies had a seven-plus score (Table 1), guaranteeing the eligibility for meta-analysis.

**TABLE 1:** Summary of studies included in the meta-analysis.

Study	Country	Nature of study	Initial infection	Sample size (*n* =)		Age at onset(months)		Risk factors considered	Study quality (NOS)
				PIBO group	Control group	PIBO group	Control group		
Yu et al. (13)	China	Retrospective	Severe ADV pneumonia	20	46	16.5 (11,25.25) *	30.5 (17, 50.75)	Male, premature birth, a history of wheezing, wheezing, hypoxemia, pulmonary consolidation, atelectasis, pleural effusion, co-infection of bacteria, use of glucocorticoids, use of γ-globulin, mechanical ventilation	8
Lee et al. (14)	Korea	Retrospective	MPP	18	132	57.6 ± 31.2	73.2 ± 46.8	Age, male, allergic rhinitis	8
Colom et al. (15)	Argentina	Case-control	Bronchiolitis	109	99	6 (1–26)**	5 (1–20)	Male, mechanical ventilation	9
Castro-Rodriguez et al. (16)	Chile	Prospective	ADV pneumonia	18	20	12.5 ± 10.3	13.4 ± 15.5	Age, male, rhinitis, a history of wheezing, wheezing, tachypnea, mechanical ventilation, use of glucocorticoids	8
Li et al. (17)	China	Nested case-control	ADV pneumonia	29	73	27.7 ± 19.5	40.9 ± 21.1	Age, male, allergic rhinitis, wheezing, tachypnea, pulmonary consolidation, atelectasis, pleural effusion, co-infection of bacteria	9
Dai et al. (18)	China	Case-control	ADV pneumonia	37	229	12 (8, 17.5)*	32 (13, 48)	Male, premature birth, a history of wheezing, wheezing, tachypnea, hypoxemia, co-infection of bacteria	8
Li et al. (19)	China	Retrospective	ADV pneumonia	98	108	18.28 ± 15.17	26.98 ± 28.72	Age, male, a history of wheezing	8
Wu et al. (20)	China	Retrospective	ADV infection	14	530	15.5 (6–72)**	23.5 (1–144)	Male, premature birth, use of glucocorticoids, use of γ-globulin, mechanical ventilation	8
Zhong et al. (21)	China	Retrospective	Severe ADV pneumonia	34	105	15.1 (7.2)***	20.5 (14.6)	Male, premature birth, pulmonary consolidation, atelectasis, pleural effusion, co-infection of bacteria, use of glucocorticoids, use of γ-globulin, mechanical ventilation	8

*Data are shown as median (Q1 and Q3).

**Data are shown as median (range).

***Data are shown as median (interquartile range).

*PIBO, post-infectious bronchiolitis obliterans; NOS, Newcastle–Ottawa Scale; ADV, adenovirus; MPP, mycoplasma pneumoniae pneumonia.*

*Age of the sample is reported as mean (SD) and range, when reported.*

**TABLE 2:** Summary of risk factors for post-infectious bronchiolitis obliterans (PIBO).

Risk factors		Number of studies	Odd ratio (95% CI)	*p*-Value	Effect model	Heterogeneity		
						Chi-squared	*p*-Value	*I*^2^ (%)
Male		9	1.52 (1.14, 2.01)	0.004	F	3.96	0.86	0
Premature birth		4	1.50 (0.66, 3.43)	0.330	F	3.49	0.32	14
Comorbidities	A history of wheezing	4	2.22 (1.36, 3.63)	0.002	F	2.38	0.50	0
	Allergic rhinitis	3	1.60 (0.38, 6.70)	0.520	R	9.86	0.007	80
Clinical manifestations	Wheezing	4	7.73 (2.73, 21.93)	0.000	R	8.26	0.04	64
	Tachypnea	3	10.14 (2.66, 38.74)	0.000	R	9.56	0.008	79
	Hypoxemia	2	21.54 (10.00, 46.36)	0.000	F	0.01	0.92	0
Imaging findings	Pulmonary consolidation	3	1.20 (0.66, 2.19)	0.550	F	3.76	0.15	47
	Atelectasis	3	1.25 (0.46, 3.38)	0.670	R	4.89	0.09	59
	Pleural effusion	3	0.65 (0.34, 1.25)	0.200	F	1.32	0.52	0
Co-infection of bacteria		4	2.23 (1.40, 3.53)	0.000	F	3.40	0.32	12
Treatment	Use of glucocorticoids	4	4.46 (1.26, 15.79)	0.020	R	6.90	0.03	71
	Use of γ-globulin	3	4.77 (2.34, 9.73)	0.000	F	2.16	0.34	7
	Mechanical ventilation	5	14.61 (7.53, 28.35)	0.000	F	0.56	0.91	0

*CI, confidence interval; R, random; F, fixed.*

### Age

**FIGURE 2:**
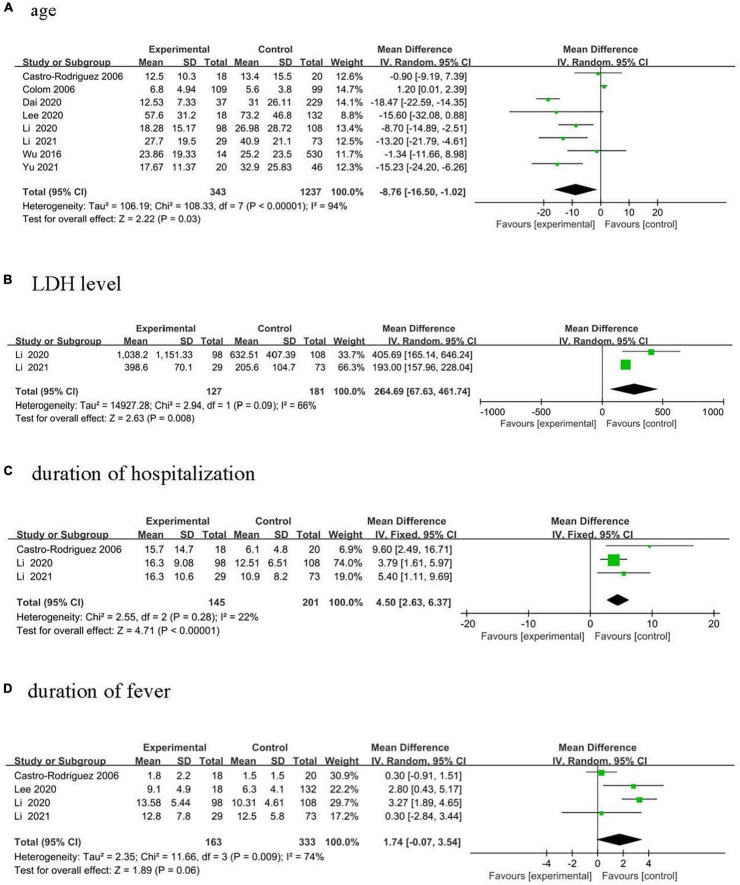
Forest plots of MD estimates for the following risk factors: **(A)** age; **(B)** LDH level; **(C)** duration of hospitalization; and **(D)** duration of fever.

Eight studies (five studies from China and one each from Argentina, Korea, and Chile), were included, comprising 343 cases and 1,237 controls, to study the association between age and PIBO from 1991 to 2020 (13–20). Cases that developing PIBO had a lower age compared with controls as shown in Figure 2A (MD, −8.76 months, 95% CI: −16.50 to −1.02, *p* = 0.003).

### Male

Nine studies (three studies from China and one each from Argentina, Korea, and Chile) were included, consisting of 377 cases and 1,342 controls, to study the association between being male and PIBO from 1991 to 2020 (13–21). Meta-analysis calculated a pooled OR of 1.52 (95% CI: 1.14–2.01).

### Hypoxemia

Two studies from China were included, comprising 57 cases and 275 controls, to analyze the association between hypoxemia and PIBO from 2011 to 2020 (13, 18). Meta-analysis calculated a pooled OR of 21.54 (95% CI: 10–46.36).

### Mechanical Ventilation

Five studies (three studies from China and one each from Argentina and Chile), were included, comprising 195 cases and 800 controls, to study the association between mechanical ventilation and PIBO from 1991 to 2020 (13, 15, 16, 20, 21). Meta-analysis calculated a pooled OR of 14.61 (95% CI: 7.53–28.35).

### Tachypnea

Three studies (two studies from China and one from Chile) were included, comprising 84 cases and 322 controls, to study the association between tachypnea and PIBO from 1998 to 2020 (16–18). Meta-analysis calculated a pooled OR of 10.14 (95% CI: 2.66–38.74).

### Wheezing

Four studies were included (three studies from China and one from Chile), consisting of 104 cases and 368 controls, to study the association between wheezing and PIBO from 1998 to 2020 (13, 16–18). Meta-analysis calculated a pooled OR of 7.73 (95% CI: 2.73–21.93).

### Use of γ-Globulin

Three studies from China were included, consisting of 58 cases and 681 controls, to study the association between the use of γ-globulin and PIBO from 2011 to 2020 (13, 20, 21). Meta-analysis calculated a pooled OR of 4.77 (95% CI: 2.34–9.73).

### Use of Glucocorticoids

Four studies (three studies from China and one from Chile) were included, consisting of 86 cases and 701 controls, to study the association between the use of glucocorticoids and PIBO from 1998 to 2020 (13, 16, 20, 21). Meta-analysis calculated a pooled OR of 4.46 (95% CI: 1.26–15.79).

### Co-infection of Bacteria

Four studies from China were included, consisting of 120 cases and 453 controls, to study the association between co-infection of bacteria and PIBO from 2011 to 2020 (13, 17, 20, 21). Meta-analysis calculated a pooled OR of 2.23 (95% CI: 1.40–3.53).

### A History of Wheezing

Four studies (six studies from China and one from Chile) were included, consisting of 173 cases and 403 controls, to study the association between a history of wheezing and PIBO from 1998 to 2020 (13, 16, 18, 19). Meta-analysis calculated a pooled OR of 2.22 (95% CI: 1.36–3.63).

### LDH Levels

Two studies from China were included, comprising 127 cases and 181 controls, to analyze the association between LDH levels and PIBO from 2012 to 2020 (17, 19). Children diagnosed with PIBO had higher LDH levels (Figure 2B, MD, 264.69 U/L, 95% CI: 67.43–461.74, *p* = 0.008).

### Duration of Hospitalization

Three studies from China were included, comprising 145 cases and 201 controls, to analyze the association between duration of hospitalization and PIBO from 1998 to 2020 (16, 17, 19). Children diagnosed with PIBO had a higher duration of hospitalization (Figure 2C, MD, 4.5 days, 95% CI: 2.63–6.37, *p* < 0.001).

### Other Potential Factors Require Further Confirmation

Three studies evaluated lung imaging evidence (13, 17, 21). Meta-analysis calculated a pooled OR respectively, pulmonary consolidation of 1.20 (95% CI: 0.66–2.19, *p* = 0.55), atelectasis of 1.25 (95% CI: 0.46–3.38, *p* = 0.67), pleural effusion of 0.65 (95% CI: 0.34–1.25, *p* = 0.20). No significant differences were observed in Premature birth (pooled OR 1.50, 95% CI: 066–3.43, *p* = 0.33) (13, 18, 20, 21), Allergic rhinitis (pooled OR 1.60, 95% CI: 0.38–6.70, *p* = 0.52) (14, 16, 17), and duration of fever (Figure 2D, MD = 1.74 days, 95% CI: −0.07 to 3.54, *p* = 0.06) (14, 16, 17, 19).

## Discussion

This is the first comprehensive systematic review and meta-analysis to date, which attempt to systematically assess the effect of a multitude of possible risk factors on PIBO in children. We identified 14 risk factors in total. This meta-analysis was the first to show that hypoxemia was most strongly associated with PIBO (OR 21.54). Mechanical ventilation was found to confer a significant risk (OR 14.61). Clinical manifestations, such as tachypnea and wheezing, were also observed to be prominent risk factors (OR 10.14 and 7.73, respectively). Use of γ-globulin (OR 4.77), use of glucocorticoids (OR 4.46), co-infection of bacteria (OR 2.23), a history of wheezing (OR 2.22) and being male (OR 1.52) were other prominent risk factors. The effects of premature birth, allergic rhinitis, duration of fever, and imaging finding (pulmonary consolidation, atelectasis, and pleural effusion) are less clear and require further confirmation. Cases that developing PIBO had a lower age compared with controls (MD, −8.76 months, 95% CI: −16.50 to −1.02). Both LDH levels and duration of hospitalization had a statistically significant association with PIBO (MD, 264.69 U/L, 95% CI: 67.43 to 461.74 and MD, 4.50 days, 95% CI: 2.63 to 6.37, respectively). This is the first meta-analysis to examine the association between hypoxemia, mechanical ventilation, tachypnea, and wheezing with PIBO.

In this meta-analysis, we found that hypoxemia was the strongest predictor of PIBO, which was a novel finding. Hypoxemia usually occurs earlier than mechanical ventilation, which made it a more sensitive and good early warning indicator than other predictors. No mechanisms have been proposed to explain the adverse effects of hypoxia on the development of PIBO. Based on the limited data shown in animal studies, Matthew and colleagues showed the development of BO and pulmonary fibrosis 1 month after sulfur mustard (SM) inhalation exposure in rats (22). They found that respiratory distress developed over time after SM inhalation, with progressive hypoxemia, respiratory distress, and weight loss. TGF-b1 and PDGF, two profibrotic cytokines and pathways, remained chronically elevated in rat lungs. The role of hypoxemia needs to be further investigated in animal models in the future.

Although our meta-analysis found that mechanical ventilation was a significant risk factor for PIBO, our results do not indicate that mechanical ventilation causes injury to the lung that increases the risk of developing PIBO, or as an indicator of disease severity. Further studies are needed to elucidate the relationship between mechanical ventilation and PIBO and to investigate the need for lung-protective strategies in this susceptible population (15).

Hypoxemia and mechanical ventilation were associated with severe lung injury. Based on the results of the meta-analysis, we speculated that predictors of PIBO showed that factors representing acute pulmonary insult severity were correlated with PIBO development, and it seemed that the more severe the episode of initial pneumonia, the higher the incidence of PIBO.

Most patients with PIBO are younger than 3 years of age at onset. Huang calculated that the median age of the 56 children with PIBO was 17.71 (7–91) months (23). Hong Kong’s 20-year single-center research data show that the median age at diagnosis was 1.39 years (IQR: 0.84–4.99 years), 26 patients were included with a male predominance (72.2%) (24). Our meta-analysis showed that younger children and males were at higher risk of PIBO than older children. Therefore, careful attention should be paid especially to younger male children.

In our study, specific clinical manifestations such as tachypnea and wheezing were significant risk factors of PIBO. The patient usually presents wheezing, tachypnea, dyspnea, and persistent cough for weeks or months after the initial infection (25–27). Yazan et al. evaluated 114 children with PIBO and found that persistent wheezing was the most common complaint (28). The typical ventilatory pattern of the PIBO disease is a severe ventilatory obstruction, which often does not respond to the treatments administered (29). After the initial attack, the disease can persist for years. Jerkic et al. demonstrated that in patients with PIBO, pulmonary function decreased significantly showing persistent obstruction over an average follow-up period of 8 years (30).

Our analysis also confirms that the association between several initial imaging factors and PIBO has a very small effect. These include pulmonary consolidation, atelectasis, and pleural effusion. The most common HRCT finding is the mosaic pattern in children with PIBO (31–33). It is not clear if CT changes significantly progress to deterioration as children grow up (34).

Our study also found that a history of wheezing was significantly associated with developing PIBO compared with allergic rhinitis. The number of studies included was small, and we did not assess the relationship between atopic dermatitis, history of eczema, allergic sensitization early in life, allergic sensitization early in life, tobacco, and PIBO. It appears that more well-designed clinical studies are needed to identify risk factors of PIBO.

Although this study has summarized the major risk factors for the development of PIBO, our systematic review has also limitations. The timing of assessment of the clinical manifestations and treatment of disease was generally not available. The definition and method of summarizing risk factors, the method of analysis, and the method of reporting results were heterogeneous. Given the limited number of studies included in the analysis, the findings from our meta-analysis should be confirmed in future research. One aim of the study was to precisely quantify the risk associated with these factors by combining center estimates in a meta-analysis. To pursue this aim, an agreement to supply and combine common data from many centers is required. This might be achieved through a special working group in the provision of the relevant data to central registries for analysis. Fortunately, since 2016, an international consortium of experts consisting of pediatric pulmonologists, radiologists, pathologists, physical therapists, psychologists, basic scientists, and statisticians has gathered regularly for a workshop on PIBO in Geisenheim, Germany (35). Further multicenter prospective trials are required to understand the complexity of the disease and to define risk factors.

In future studies, additional analyses are required to test other factors that may also be predictive of PIBO in children. Furthermore, studies should investigate the role of hypoxemia and another possible case mechanism by studying larger samples and by more careful measurement and analysis of possible confounding factors. Further multicenter prospective trials are required to understand the complexity of the disease and to define risk factors.

## Conclusion

In conclusion, this systematic review and meta-analysis have found hypoxemia to be the most significant risk factor for PIBO, followed by mechanical ventilation, tachypnea, and wheezing. Use of glucocorticoids, use of γ-globulin, co-infection of bacteria, a history of wheezing, and being male may also play a role. The factors identified by our meta-analysis can inform the generation of a clinical prediction model for the developing PIBO in children. Further investigation is required to verify this relationship and explore other aspects of the aetiopathogenesis of PIBO.

## Data Availability Statement

The original contributions presented in the study are included in the article/Supplementary Material, further inquiries can be directed to the corresponding author.

## Author Contributions

DL, JL, and QZ designed the study. JL contributed to the literature search, data collection, statistical analysis, and drafting of the manuscript. DL, LZ, and YC contributed to the literature search and data collection. QZ performed the manuscript review. All authors have read and approved the content of the final manuscript.

## Conflict of Interest

The authors declare that the research was conducted in the absence of any commercial or financial relationships that could be construed as a potential conflict of interest.

## Publisher’s Note

All claims expressed in this article are solely those of the authors and do not necessarily represent those of their affiliated organizations, or those of the publisher, the editors and the reviewers. Any product that may be evaluated in this article, or claim that may be made by its manufacturer, is not guaranteed or endorsed by the publisher.
