# Supplementary material for: Risk Factors for Post-infectious Bronchiolitis Obliterans in Children: A Systematic Review and Meta-Analysis
Source: Front Pediatr. 2022 Jun 9;10:881908. doi: 10.3389/fped.2022.881908 (PMC9218415; doi:10.3389/fped.2022.881908)
Supplement: Supplementary file 1 [file Data_Sheet_1.pdf]

Supplementary Material

Search strategy:

PubMed– 113 results

(((((((("Adolescent"[MeSH Terms] OR ("Adolescents"[Title/Abstract] OR "Adolescence"[Title/Abstract] OR "Teens"[Title/Abstract] OR "Teen"[Title/Abstract] OR "Teenagers"[Title/Abstract] OR "Teenager"[Title/Abstract] OR "Youth"[Title/Abstract] OR "Youths"[Title/Abstract] OR "adolescents female"[Title/Abstract] OR "adolescent female"[Title/Abstract] OR "female adolescent"[Title/Abstract] OR "female adolescents"[Title/Abstract] OR "adolescents male"[Title/Abstract] OR "adolescent male"[Title/Abstract] OR "male adolescent"[Title/Abstract] OR "male adolescents"[Title/Abstract]) OR ("Child"[MeSH Terms] OR "Children"[Title/Abstract]) OR ("child, preschool"[MeSH Terms] OR ("preschool child"[Title/Abstract] OR "children preschool"[Title/Abstract] OR "preschool children"[Title/Abstract])) OR ("Infant"[MeSH Terms] OR "Infants"[Title/Abstract])) AND ("Bronchiolitis Obliterans"[MeSH Terms] OR ("constrictive bronchiolitis"[Title/Abstract] OR (("Bronchiolitis"[MeSH Terms] OR "Bronchiolitis"[All Fields] OR "Bronchiolitides"[All Fields]) AND "Constrictive"[Title/Abstract]) OR "bronchiolitis constrictive"[Title/Abstract] OR (("constrict"[All Fields] OR "constricted"[All Fields] OR "constricting"[All Fields] OR "constriction"[MeSH Terms] OR "constriction"[All Fields] OR "constrictions"[All Fields] OR "constriction, pathologic"[MeSH Terms] OR ("constriction"[All Fields] AND "pathologic"[All Fields]) OR "pathologic constriction"[All Fields] OR "Constrictive"[All Fields] OR "constricts"[All Fields]) AND "Bronchiolitides"[Title/Abstract]) OR (("Bronchiolitis"[MeSH Terms] OR "Bronchiolitis"[All Fields] OR "Bronchiolitides"[All Fields]) AND "Exudative"[Title/Abstract]) OR (("Bronchiolitis"[MeSH Terms] OR "Bronchiolitis"[All Fields] OR "Bronchiolitides"[All Fields]) AND "Exudative"[Title/Abstract]) OR (("exudated"[All Fields] OR "exudates and transudates"[MeSH Terms] OR ("exudates"[All Fields] AND "transudates"[All Fields]) OR "exudates and transudates"[All Fields] OR "exudate"[All Fields] OR "exudates"[All Fields] OR "exudating"[All Fields] OR "exudation"[All Fields] OR "exudations"[All Fields] OR "Exudative"[All Fields] OR "exude"[All Fields] OR "exuded"[All Fields] OR "exudes"[All Fields] OR "exuding"[All Fields]) AND "Bronchiolitides"[Title/Abstract]) OR "exudative bronchiolitis"[Title/Abstract] OR "bronchiolitis proliferative"[Title/Abstract] OR (("Bronchiolitis"[MeSH Terms] OR "Bronchiolitis"[All Fields] OR "Bronchiolitides"[All Fields]) AND "Proliferative"[Title/Abstract]) OR (("Proliferative"[All Fields] OR "proliferatively"[All Fields] OR "proliferatives"[All Fields]) AND "Bronchiolitides"[Title/Abstract]) OR "proliferative bronchiolitis"[Title/Abstract])) AND 1988/01/01:2022/12/31[Date - Publication]) NOT ((("Transplantation"[Title/Abstract] OR "Transplantations"[Title/Abstract] OR "Transplant OR"[Title/Abstract]) AND "Transplants"[Title/Abstract]) OR "organizing pneumonia"[Title/Abstract] OR "Stevens-Johnson Syndrome"[Title/Abstract] OR "interstitial lung disease"[Title/Abstract] OR "bronchiectasis"[Title/Abstract] OR "case report"[Title/Abstract])) NOT "Case Reports"[Publication Type]) NOT "Review"[Publication Type]) NOT ("Cystic Fibrosis"[MeSH Terms] OR ("fibrosis cystic"[Title/Abstract] OR "Mucoviscidosis"[Title/Abstract] OR "pulmonary cystic fibrosis"[Title/Abstract] OR "cystic fibrosis pulmonary"[Title/Abstract] OR "pancreatic cystic

fibrosis"[Title/Abstract] OR "cystic fibrosis pancreatic"[Title/Abstract] OR "fibrocystic disease of pancreas"[Title/Abstract] OR "pancreas fibrocystic disease"[Title/Abstract] OR (("pancrea"[All Fields] OR "Pancreas"[MeSH Terms] OR "Pancreas"[All Fields]) AND "fibrocystic diseases"[Title/Abstract]) OR "cystic fibrosis of pancreas"[Title/Abstract]))) NOT ("bronchiectasis"[MeSH Terms] OR "Bronchiectases"[Title/Abstract])) NOT ("lung diseases, interstitial"[MeSH Terms] OR "Interstitial Lung Diseases"[Title/Abstract])) NOT "Stevens-Johnson Syndrome"[MeSH Terms]) NOT ("Bone Marrow Transplantation"[MeSH Terms] OR "Hematopoietic Stem Cell Transplantation"[MeSH Terms] OR "Heart-Lung Transplantation"[MeSH Terms] OR "lung Transplantation"[MeSH Terms])

No.	Query	Results
#6	#5 NOT ('transplantation':ab,ti OR 'transplantations':ab,ti OR 'transplant':ab,ti OR 'transplants':ab,ti OR 'organizing pneumonia':ab,ti OR 'stevens–johnson syndrome':ab,ti OR 'interstitial lung disease':ab,ti OR 'bronchiectasis':ab,ti OR 'case report':ab,ti)	**258**
#5	#3 AND ([adolescent]/lim OR [child]/lim OR [embryo]/lim OR [fetus]/lim OR [infant]/lim OR [newborn]/lim OR [preschool]/lim OR [school]/lim) AND ('case control study'/de OR 'clinical article'/de OR 'clinical study'/de OR 'cohort analysis'/de OR 'comparative study'/de OR 'controlled study'/de OR 'cross sectional study'/de OR 'human'/de OR 'longitudinal study'/de OR 'multicenter study'/de OR 'observational study'/de OR 'prospective study'/de OR 'retrospective study'/de) AND 'article'/it	**909**
#4	#3 AND ([adolescent]/lim OR [child]/lim OR [embryo]/lim OR [fetus]/lim OR [infant]/lim OR [newborn]/lim OR [preschool]/lim OR [school]/lim)	**1515**
#3	#1 OR #2	**10157**
#2	'constrictive bronchiolitis':ab,ti OR 'bronchiolitides, constrictive':ab,ti OR 'bronchiolitis, constrictive':ab,ti OR 'constrictive bronchiolitides':ab,ti OR 'bronchiolitis, exudative':ab,ti OR 'bronchiolitides, exudative':ab,ti OR 'exudative bronchiolitides':ab,ti OR 'exudative bronchiolitis':ab,ti OR 'bronchiolitis, proliferative':ab,ti OR 'bronchiolitides, proliferative':ab,ti OR 'proliferative bronchiolitides':ab,ti OR 'proliferative bronchiolitis':ab,ti	**326**

#1	'bronchiolitis obliterans'/exp OR 'bronchiolitis obliterans'	**10094**

Embase – **258 results**

Web of Science:

#1 TS=(Bronchiolitis Obliterans OR Constrictive Bronchiolitis OR Bronchiolitides, Constrictive OR Bronchiolitis, Constrictive OR Constrictive Bronchiolitides OR Bronchiolitis, Exudative OR Bronchiolitides, Exudative OR Exudative Bronchiolitides OR Exudative Bronchiolitis OR Bronchiolitis, Proliferative OR Bronchiolitides, Proliferative OR Proliferative Bronchiolitides OR Proliferative Bronchiolitis)

#2 TS=(Adolescent OR Adolescents OR Adolescence OR Teens OR Teen OR Teenagers OR Teenager OR Youth OR Youths OR Adolescents, Female OR Adolescent, Female OR Female Adolescent OR Female Adolescents OR Adolescents, Male OR Adolescent, Male OR Male Adolescent OR Male Adolescents OR child OR Children OR Child, PreschooL OR Preschool Child OR Children, Preschool OR Preschool Children OR infant OR infants)

#3 #1AND #2

#4 #3NOT TS =(Transplantation OR Transplantations OR Transplant OR Transplants OR organizing pneumonia OR Stevens-Johnson Syndrome OR interstitial lung disease OR bronchiectasis OR case report)

Supplementary Figures

Supplementary Figure 1. Forest plot showing pooled analyses of hypoxemia and PIBO.

Supplementary Figure 2. Forest plot showing pooled analyses of mechanical ventilation and PIBO.

Supplementary Figure 3. Forest plot showing pooled analyses of tachypnea and PIBO.

Supplementary Figure 4. Forest plot showing pooled analyses of wheezing and PIBO.

Supplementary Figure 5. Forest plot showing pooled analyses of use of γ-globulin and PIBO.

Supplementary Figure 6. Forest plot showing pooled analyses of use of glucocorticoids and PIBO.

Supplementary Figure 7. Forest plot showing pooled analyses of co-infection of bacteria and PIBO.

Supplementary Figure 8. Forest plot showing pooled analyses of a history of wheezing and PIBO.

Supplementary Figure 9. Forest plot showing pooled analyses of male and PIBO.

Supplementary Figure 10. Forest plot showing pooled analyses of premature birth and PIBO.

Supplementary Figure 11. Forest plot showing pooled analyses of allergic rhinitis and PIBO.

Supplementary Figure 12. Forest plot showing pooled analyses of pulmonary consolidation and PIBO.

Supplementary Figure 13. Forest plot showing pooled analyses of atelectasis and PIBO.

Supplementary Figure 14. Forest plot showing pooled analyses of pleural effusion and PIBO.

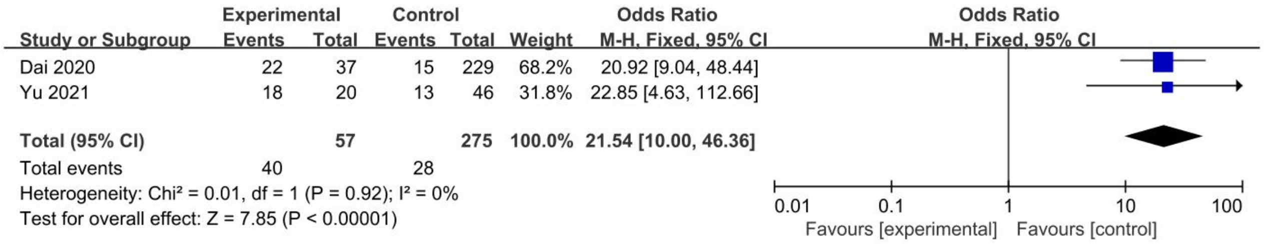

Supplementary Figure 1. Forest plot showing pooled analyses of hypoxemia and PIBO.

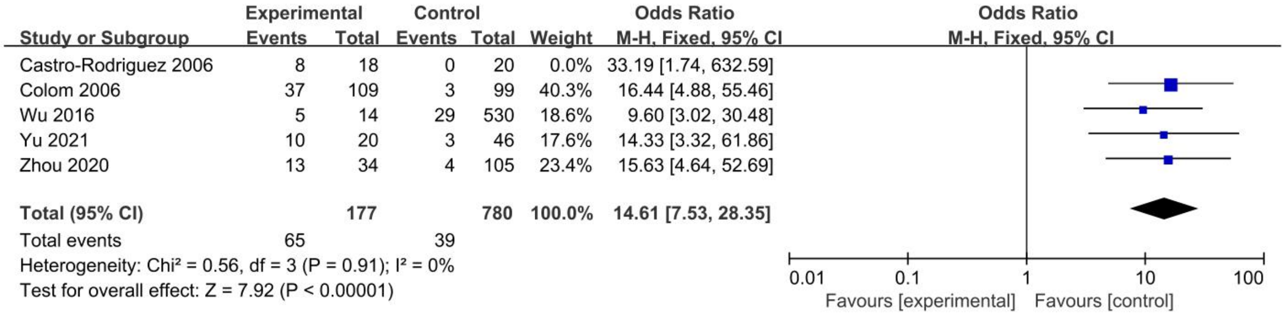

Supplementary Figure 2. Forest plot showing pooled analyses of mechanical ventilation and PIBO.

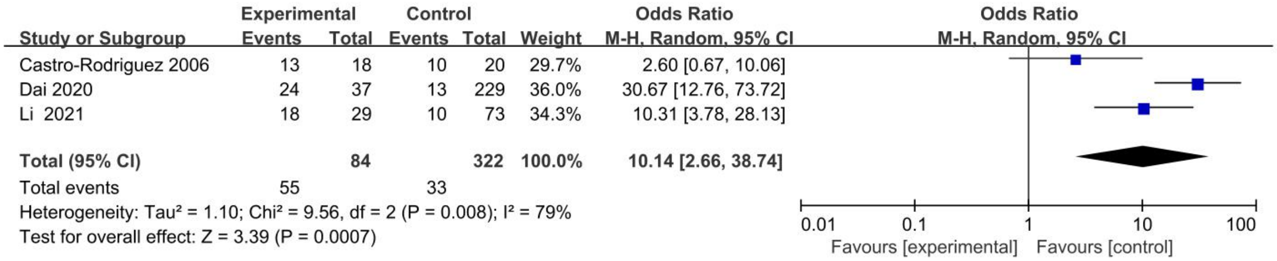

Supplementary Figure 3. Forest plot showing pooled analyses of tachypnea and PIBO.

Study or Subgroup	Experimental Events	Experimental Total	Control Events	Control Total	Weight	Odds Ratio M-H, Random, 95% CI
Castro-Rodriguez 2006	13	18	12	20	24.1%	1.73 [0.44, 6.79]
Dai 2020	30	37	64	229	31.9%	11.05 [4.62, 26.42]
Li 2021	24	29	30	73	28.7%	6.88 [2.36, 20.07]
Yu 2021	19	20	13	46	15.3%	48.23 [5.84, 398.14]
**Total (95% CI)**		**104**		**368**	**100.0%**	**7.73 [2.73, 21.93]**
Total events	86		119			

Heterogeneity: Tau² = 0.69; Chi² = 8.26, df = 3 (P = 0.04); I² = 64%
Test for overall effect: Z = 3.85 (P = 0.0001)

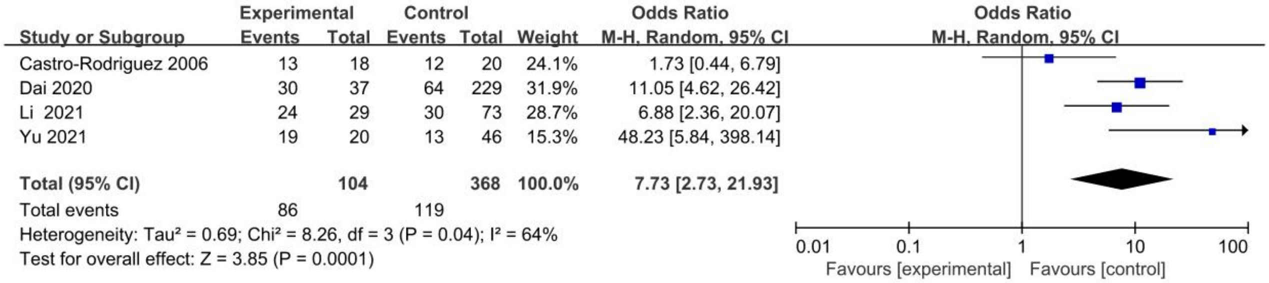

Supplementary Figure 4. Forest plot showing pooled analyses of wheezing and PIBO.

Study or Subgroup	Experimental Events	Experimental Total	Control Events	Control Total	Weight	Odds Ratio M-H, Fixed, 95% CI
Wu 2016	12	14	195	530	16.7%	10.31 [2.28, 46.54]
Yu 2021	17	20	33	46	34.9%	2.23 [0.56, 8.92]
Zhong 2020	29	34	58	105	48.5%	4.70 [1.69, 13.09]
**Total (95% CI)**		**68**		**681**	**100.0%**	**4.77 [2.34, 9.73]**
Total events	58		286			

Heterogeneity: Chi² = 2.16, df = 2 (P = 0.34); I² = 7%
Test for overall effect: Z = 4.30 (P < 0.0001)

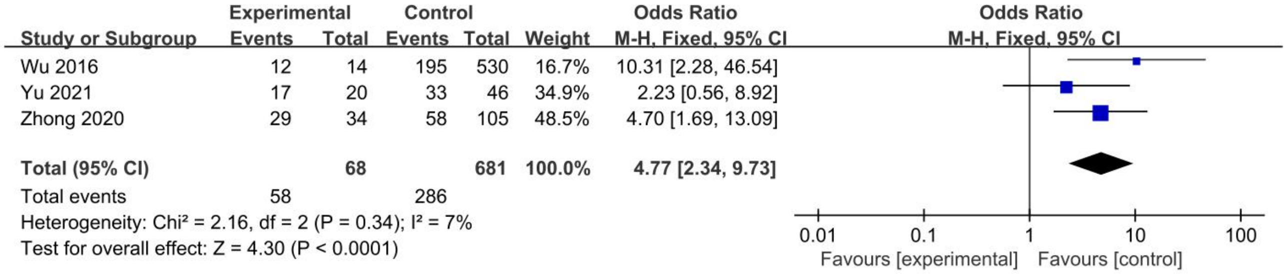

Supplementary Figure 5. Forest plot showing pooled analyses of use of γ-globulin and PIBO.

Study or Subgroup	Experimental Events	Experimental Total	Control Events	Control Total	Weight	Odds Ratio M-H, Random, 95% CI
Castro-Rodriguez 2006	15	18	9	20	28.1%	6.11 [1.34, 27.96]
Wu 2016	11	14	129	530	31.7%	11.40 [3.13, 41.49]
Zhou 2020	16	34	36	105	40.1%	1.70 [0.78, 3.73]
**Total (95% CI)**		**66**		**655**	**100.0%**	**4.46 [1.26, 15.79]**
Total events	42		174			

Heterogeneity: Tau² = 0.88; Chi² = 6.90, df = 2 (P = 0.03); I² = 71%
Test for overall effect: Z = 2.32 (P = 0.02)

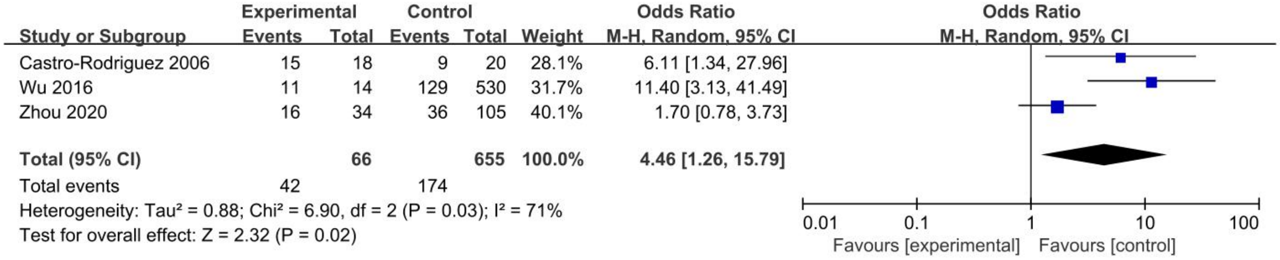

Supplementary Figure 6. Forest plot showing pooled analyses of use of glucocorticoids and PIBO.

Study or Subgroup	Experimental Events	Experimental Total	Control Events	Control Total	Weight	Odds Ratio M-H, Fixed, 95% CI
Dai 2020	14	37	38	229	28.4%	3.06 [1.45, 6.48]
Li 2021	7	29	12	73	22.4%	1.62 [0.56, 4.63]
Yu 2021	5	20	12	46	23.6%	0.94 [0.28, 3.16]
Zhou 2020	27	34	59	105	25.7%	3.01 [1.20, 7.52]
**Total (95% CI)**		**120**		**453**	**100.0%**	**2.23 [1.40, 3.53]**
Total events	53		121			

Heterogeneity: Chi² = 3.40, df = 3 (P = 0.33); I² = 12%
Test for overall effect: Z = 3.39 (P = 0.0007)

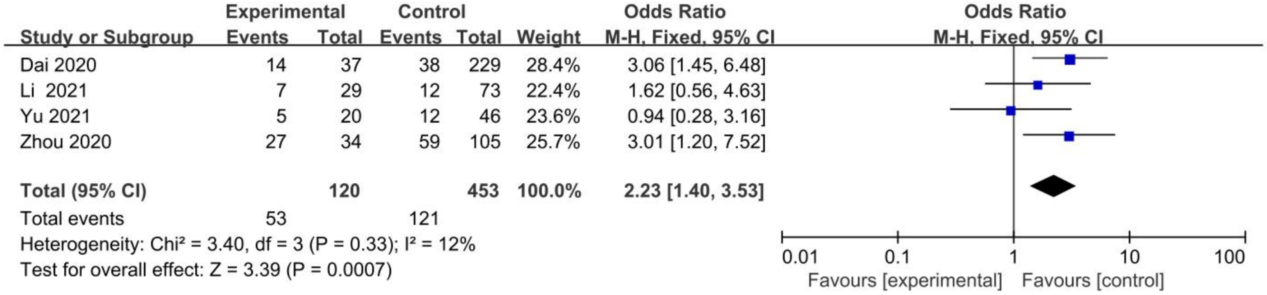

Supplementary Figure 7. Forest plot showing pooled analyses of co-infection of bacteria and PIBO.

	Experimental		Control			Odds Ratio
Study or Subgroup	Events	Total	Events	Total	Weight	M-H, Fixed, 95% CI
Castro-Rodriguez 2006	16	18	16	20	8.2%	2.00 [0.32, 12.51]
Dai 2020	12	37	57	229	52.0%	1.45 [0.68, 3.07]
Li 2020	24	98	10	108	34.9%	3.18 [1.43, 7.05]
Yu 2021	3	20	2	46	5.0%	3.88 [0.60, 25.31]
**Total (95% CI)**		**173**		**403**	**100.0%**	**2.22 [1.36, 3.63]**
Total events	55		85			

Heterogeneity: Chi² = 2.38, df = 3 (P = 0.50); I² = 0%
Test for overall effect: Z = 3.17 (P = 0.002)

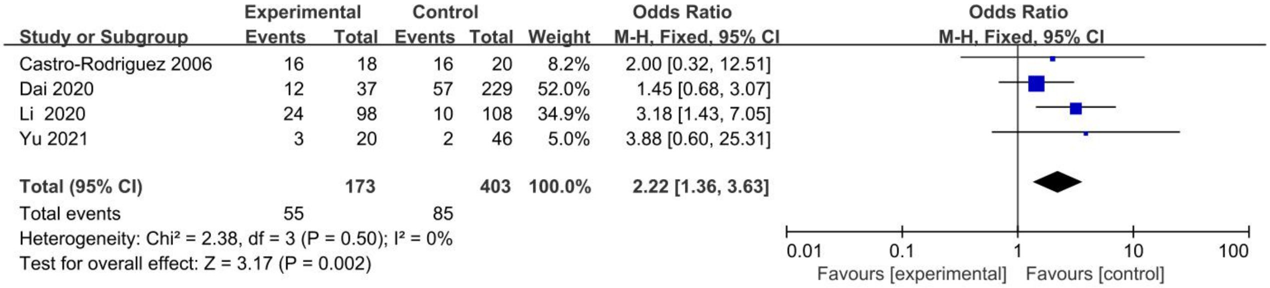

Supplementary Figure 8. Forest plot showing pooled analyses of a history of wheezing and PIBO.

	Experimental		Control			Odds Ratio
Study or Subgroup	Events	Total	Events	Total	Weight	M-H, Fixed, 95% CI
Castro-Rodriguez 2006	12	18	14	20	5.6%	0.86 [0.22, 3.37]
Colom 2006	65	109	57	99	30.4%	1.09 [0.63, 1.89]
Dai 2020	28	37	141	229	12.0%	1.94 [0.88, 4.31]
Lee 2020	11	18	67	132	7.9%	1.52 [0.56, 4.17]
Li 2020	79	98	76	108	17.7%	1.75 [0.91, 3.35]
Li 2021	23	29	51	73	7.6%	1.65 [0.59, 4.62]
Wu 2016	13	14	385	530	1.8%	4.90 [0.63, 37.76]
Yu 2021	13	20	24	46	6.4%	1.70 [0.57, 5.04]
Zhou 2020	26	34	73	105	10.6%	1.42 [0.58, 3.49]
**Total (95% CI)**		**377**		**1342**	**100.0%**	**1.52 [1.14, 2.01]**
Total events	270		888			

Heterogeneity: Chi² = 3.96, df = 8 (P = 0.86); I² = 0%
Test for overall effect: Z = 2.86 (P = 0.004)

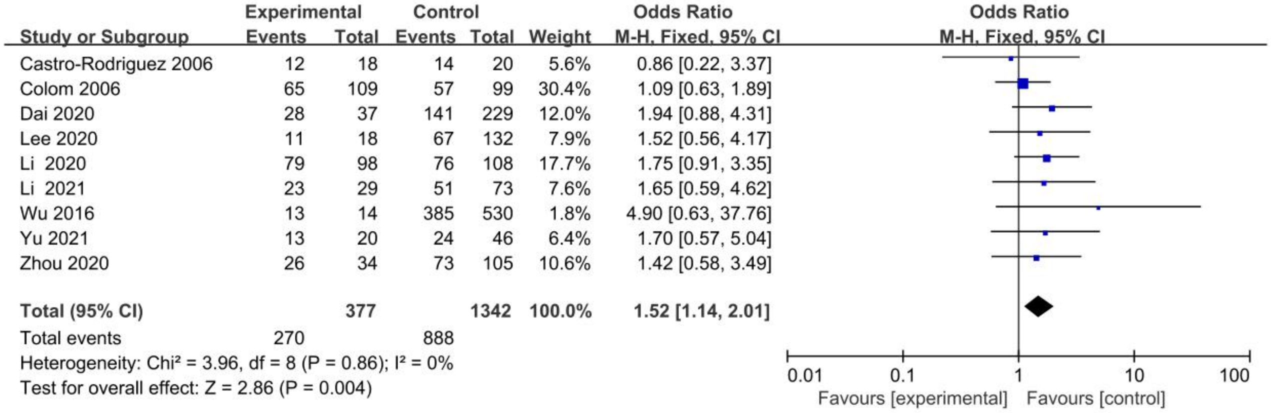

Supplementary Figure 9. Forest plot showing pooled analyses of male and PIBO.

	Experimental		Control			Odds Ratio
Study or Subgroup	Events	Total	Events	Total	Weight	M-H, Fixed, 95% CI
Dai 2020	4	37	7	229	20.9%	3.84 [1.07, 13.85]
Wu 2016	1	14	34	530	19.5%	1.12 [0.14, 8.83]
Yu 2021	2	20	3	46	19.7%	1.59 [0.24, 10.35]
Zhou 2020	1	34	7	105	39.9%	0.42 [0.05, 3.58]
**Total (95% CI)**		**105**		**910**	**100.0%**	**1.50 [0.66, 3.43]**
Total events	8		51			

Heterogeneity: Chi² = 3.49, df = 3 (P = 0.32); I² = 14%
Test for overall effect: Z = 0.97 (P = 0.33)

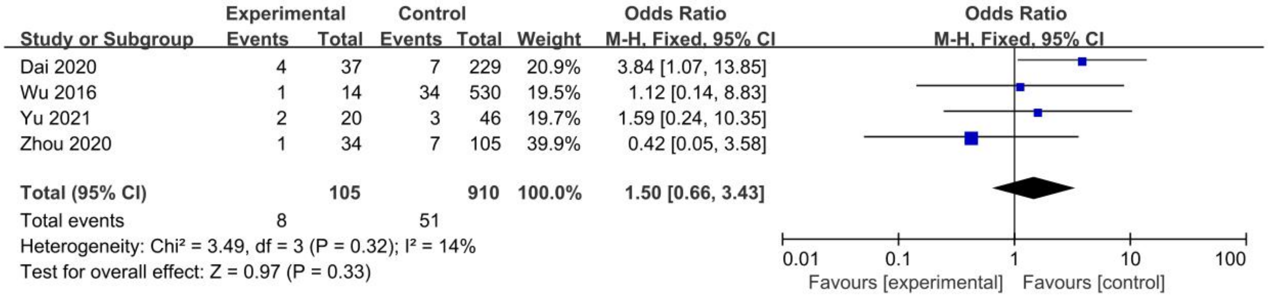

Supplementary Figure 10. Forest plot showing pooled analyses of premature birth and PIBO.

Study or Subgroup	Experimental Events	Experimental Total	Control Events	Control Total	Weight	Odds Ratio M-H, Random, 95% CI
Castro-Rodriguez 2006	5	18	11	20	30.6%	0.31 [0.08, 1.22]
Lee 2020	13	18	68	132	33.9%	2.45 [0.83, 7.25]
Li 2021	14	29	13	73	35.6%	4.31 [1.68, 11.07]
**Total (95% CI)**		**65**		**225**	**100.0%**	**1.60 [0.38, 6.70]**
Total events	32		92			

Heterogeneity: Tau² = 1.27; Chi² = 9.86, df = 2 (P = 0.007); I² = 80%
Test for overall effect: Z = 0.64 (P = 0.52)

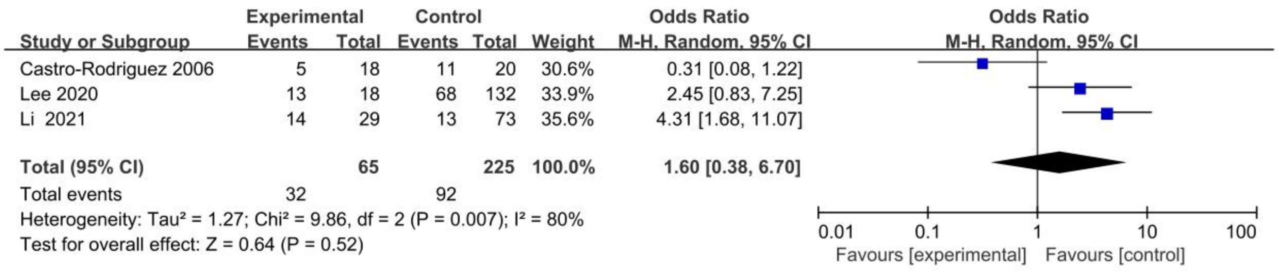

Supplementary Figure 11. Forest plot showing pooled analyses of allergic rhinitis and PIBO.

Study or Subgroup	Experimental Events	Experimental Total	Control Events	Control Total	Weight	Odds Ratio M-H, Fixed, 95% CI
Li 2021	28	29	59	73	5.9%	6.64 [0.83, 53.08]
Yu 2021	3	20	10	46	26.4%	0.64 [0.15, 2.61]
Zhou 2020	16	34	51	105	67.7%	0.94 [0.43, 2.04]
**Total (95% CI)**		**83**		**224**	**100.0%**	**1.20 [0.66, 2.19]**
Total events	47		120			

Heterogeneity: Chi² = 3.76, df = 2 (P = 0.15); I² = 47%
Test for overall effect: Z = 0.59 (P = 0.55)

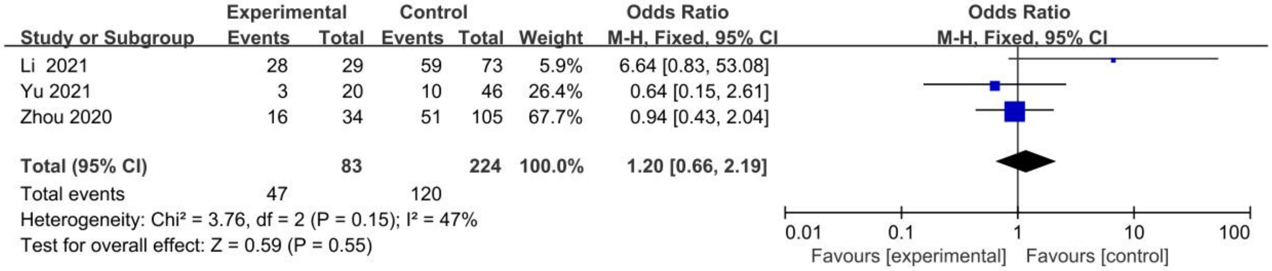

Supplementary Figure 12. Forest plot showing pooled analyses of pulmonary consolidation and PIBO.

Study or Subgroup	Experimental Events	Experimental Total	Control Events	Control Total	Weight	Odds Ratio M-H, Random, 95% CI
Li 2021	22	29	37	73	36.9%	3.06 [1.16, 8.04]
Yu 2021	5	20	13	46	31.1%	0.85 [0.26, 2.80]
Zhou 2020	4	34	18	105	32.0%	0.64 [0.20, 2.06]
**Total (95% CI)**		**83**		**224**	**100.0%**	**1.25 [0.46, 3.38]**
Total events	31		68			

Heterogeneity: Tau² = 0.46; Chi² = 4.89, df = 2 (P = 0.09); I² = 59%
Test for overall effect: Z = 0.43 (P = 0.67)

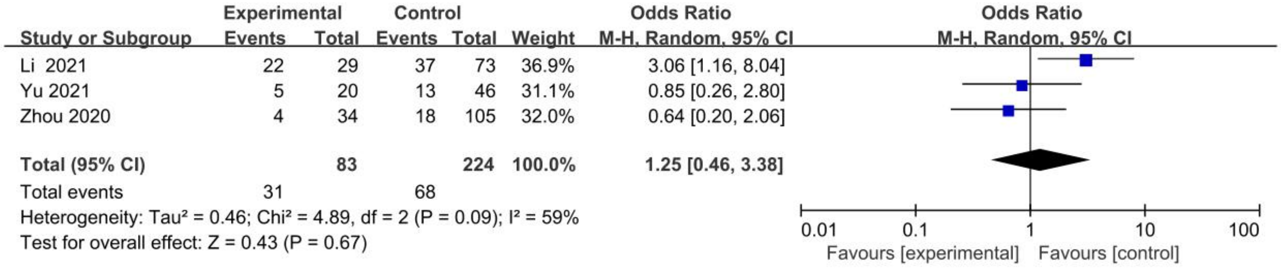

Supplementary Figure 13. Forest plot showing pooled analyses of atelectasis and PIBO.

Study or Subgroup	Experimental Events	Experimental Total	Control Events	Control Total	Weight	Odds Ratio M-H, Fixed, 95% CI
Li 2021	4	29	12	73	24.7%	0.81 [0.24, 2.76]
Yu 2021	3	20	16	46	34.6%	0.33 [0.08, 1.30]
Zhou 2020	7	34	25	105	40.7%	0.83 [0.32, 2.13]
**Total (95% CI)**		**83**		**224**	**100.0%**	**0.65 [0.34, 1.25]**
Total events	14		53			

Heterogeneity: Chi² = 1.32, df = 2 (P = 0.52); I² = 0%
Test for overall effect: Z = 1.28 (P = 0.20)

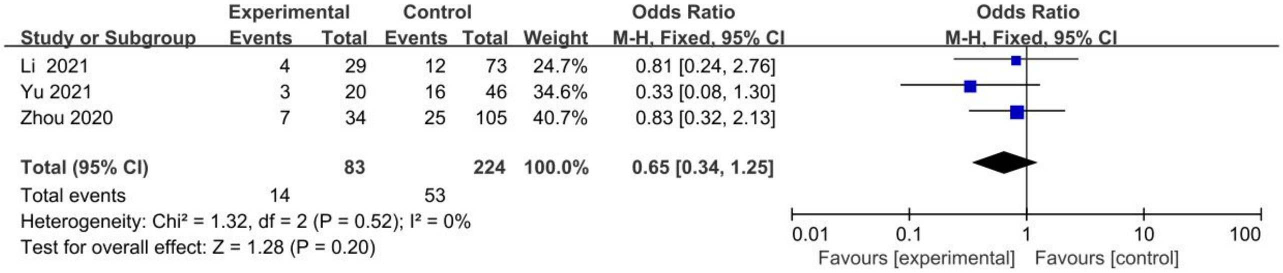

Supplementary Figure 14. Forest plot showing pooled analyses of pleural effusion and PIBO.
